# The mitogenome of freshwater loach *Homatula laxiclathra* (Teleostei: Nemacheilidae) with phylogenetic analysis of Nemacheilidae

**DOI:** 10.1002/ece3.6338

**Published:** 2020-05-13

**Authors:** Mengfei Cao, Ling Tang, Juan Chen, Xiaoyu Zhang, Russell H. Easy, Ping You

**Affiliations:** ^1^ School of Life Sciences Shaanxi Normal University Xi'an China; ^2^ Department of Biology Acadia University Wolfville NS Canada

**Keywords:** *Homatula laxiclathra*, mitogenome, Nemacheilidae, phylogenetic analysis

## Abstract

The complete mitogenome can provide valuable genetic information to reconstruct relationships between species. In this study, we sequenced a stone loach, *Homatula laxiclathra* (Teleostei: Nemacheilidae), which is found in the northern region of the Qinling Mountains in China. The size of the *H. laxiclathra* mitogenome is 16,570 bp, which contains 37 typical mitochondrial genes including 13 protein‐coding genes, 22 transfer RNAs, two ribosomal RNAs, and a control region (D‐loop) with a total AT content of 55.8%. This is similar to other Nemacheilidae sequences published in GenBank. Furthermore, a mito‐phylogenomic analysis of 46 Nemacheilidae species places *H. laxiclathra* in a robust monophyletic *Homatula* cluster with other *Homatula* species. Our results contribute toward a better understanding of a true phylogeny of these species based on large‐scale taxonomic samplings as well as to help grasp the evolution of fish mitogenomes.

## Graphical abstract

The size of *Homatula laxiclathra* mitogenome is 16,570 bp and contains 37 typical mitochondrial genes including 13 protein‐coding genes, 22 transfer RNAs, two ribosomal RNAs, and a control region (D‐loop) with the total A + T content of 55.8%, which is similar to other Nemacheilidae sequences published in GenBank.

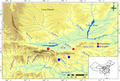

## 1 INTRODUCTION

Mitochondrial DNA can provide valuable taxon information to reconstruct evolutionary relationships between species. The fish mitogenome is circular, 15–19 k bp in size, and comprises 13 protein‐coding genes (PCGs), two ribosomal RNA genes (12S rRNA and 16S rRNA), 22 transfer RNA genes (tRANs) and two noncoding control regions (O_L_ and CR) (Miya, Kawaguchi, & Nishida, 2001). Mitogenomes are widely used for molecular systematics, phylogeography and taxa identification due to their small and simple structure, rapid evolution, maternal inheritance, and high gene conservation (Boore, 1999). In addition, molecular data for mtDNA, such as secondary structure of tRNAs and rRNAs, amino acid sequence, and codon usage can provide additional data for phylogenetic analyses (Boore, 1999; Zhu, Yan, Song, & You, 2018).

Loaches are small‐bodied freshwater fishes, which are widely distributed across Eurasia, Africa, and North America. They are popular in China due to their distinctive flavor and diverse body color. From a commercial fisheries and ornamental trade value, it is crucial to identify mtDNA mutations to avoid genetic diseases in these fish (Kipp et al., 2010). Partial mtDNA genes from the Nemacheilidae have been used for species identification and systematics (Liu et al., 2012). Unfortunately, partial mitochondrial genes do not contain complete phylogenetic information to accurately define a phylogeny (Cunha, Grande, & Zardoya, 2009; Lee, Conroy, Howel, & Kocher, 1995; Parhi, Tripathy, Priyadarshi, Mandal, & Pandey, 2019). An effective solution is to conduct comparisons of whole mtDNA from representative species of each genus (Betancur et al., 2017; Shi, Xing, Chen, Yang, & You, 2014). So far, 207 complete mitogenomes of teleostean species have been published in the GenBank database, but only 56 species from Nemacheilidae are available.

In this study, we sequenced the complete mitogenome of *Homatula laxiclathra* Gu & Zhang, 2012, which is only distributed in the northern region in Qinling Mountains. The genome structure and gene characterization of *H. laxiclathra* are compared with those reported for other *Homatula* species. To assess the deeper phylogenetic relationships of Nemacheilidaes, we reconstructed the tree using Maximum Likelihood (ML) and Bayesian inference (BI) methods. The investigation of the *H. laxiclathra* mitogenome may provide valuable evidence about teleost evolution as well as aid in species identification.

## 2 MATERIALS AND METHODS

### 2.1 Sample collection and DNA extraction

**FIGURE 1:**
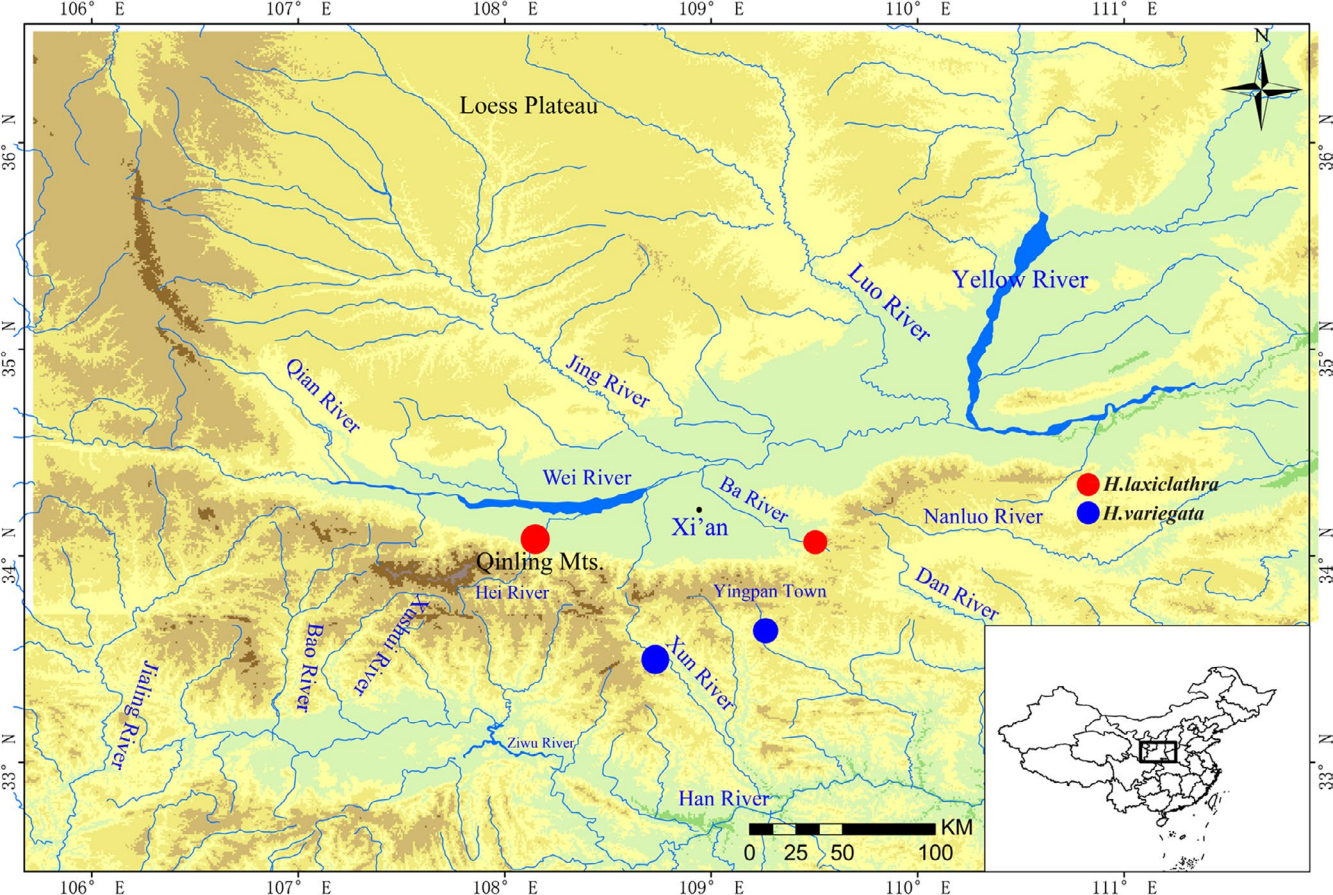
Map of sampling localities of *Homatula laxiclathra*. The map was downloaded from the Wikimedia Commons with slight modification (https://commons.wikimedia.org/wiki/File:East_Asia_topographic_map.png)

Adult specimens of *Homatula laxiclathra* Gu & Zhang, 2012 were collected from Xinguansi (33.98°N, 109.11°E), Chang'an County, the Dayu River located on the north slope of the Qinling Mountains of Shaanxi Province, Central China (Figure 1). Specimens were preserved in 95% ethanol. Animal processing was approved by the Animal Care and Use Committee of Shaanxi Normal University. Total genomic DNA was extracted from muscle tissues using a TIANamp Animal DNA Kit (Tiangen Biotech), according to the manufacturer's protocol. Voucher specimens deposited in the Fish Disease Laboratory, Shaanxi Normal University (Accession number: HL20160124).

### 2.2 PCR amplification and sequencing

Using a primer‐walking strategy, thirty conserved fish primers were designed to amplify the mitogenome (Miya & Nishida, 1999). PCR amplifications were performed with Fast*Pfu* Fly DNA polymerase (TransGen Biotech), following published PCR reaction conditions (Zhu et al., 2018).

### 2.3 Genome annotation and sequence analysis

Raw sequences were assembled using the Staden Package v1.7.0 (Staden, Beal, & Bonfield, 2000). Gene predictions were compared with published mitogenomes of *Homatula* fishes. PCGs and rRNAs were identified through DOGMA using default settings (Wyman, Jansen, & Boore, 2004). All tRNA genes and their secondary structures were verified with tRNA‐scan SE (Lowe & Eddy, 1997). The secondary structure of tRNA genes and O_L_ was drawn by RNAstructure 6.1 and modified by SturctureEditor (Mathews, 2014). MEGA 7 was used to calculate the relative synonymous codon usage (RSCU) and base composition of each gene (Kumar, Stecher, & Tamura, 2016). Nucleotide composition skew values of 13 PCGs were counted by the formulas: (AT‐skew = [A − T]/[A + T], GC‐skew = [G − C]/[G + C]) (Perna & Kocher, 1995). The complete sequence and annotation were constructed using MitoFish, including a graphic circular map (Iwasaki et al., 2013).

### 2.4 Phylogenetic analysis

A total of 43 GenBank‐retrieved mitogenomes of species from the Nemacheilidae was used to reconstruct phylogenetic relationships (Table 1). Two species from the Cyprinidae (*Hemibarbus labeo* GenBank: DQ347953, *Hemibarbus longirostris* GenBank: DQ347952) were selected as outgroups. Nucleotide sequences of 12 PCGs were aligned separately by MEGA 7 using the default setting. The ND6 gene was excluded for phylogenetic analysis due to a high degree of heterogeneity (Miya et al., 2003). The 12 PCGs were concatenated to a combination sequence without termination codon due to a high degree of degeneracy. Maximum likelihood (ML) and Bayesian inference (BI) analyses were used to define phylogenetic relationships among the Nemacheilidae (Kumar et al., 2016; Ronquist et al., 2012). The phylogenetic trees were modified by FigTree v1.4.3 (Vlad, Balaji, Vikas, Ramani, & Larry, 2008).

**TABLE 1:** Species mentioned in this study with GenBank accession number

Family	Species	Size (bp)	Whole genome composition				Accession number
			A%	C%	G%	T%	
Nemacheilidae	*Acanthocobitis botia*	16,660	30.5	26.5	15.9	27.1	AP012138
	*Acanthocobitis zonalternans*	16,642	30.1	27.1	16.5	26.4	AP012140
	*Barbatula barbatula*	16,630	28.5	27.1	18.2	26.2	KP715096
	*Barbatula nuda*	16,619	28.4	27.2	17.9	26.5	KF574248
	*Barbatula toni*	16,617	28.5	27.3	17.8	26.4	AB242162
	*Homatula potanini*	16,569	30.1	26.9	16.7	26.3	KM017732
	*Homatula variegata*	16,571	29.5	27.1	17.3	26.1	JX144893
	*Homatula laxiclathra*	16,570	29.6	27.0	17.2	26.1	MK279351
	*Lefua costata*	16,579	29.9	26.5	16.8	26.9	KT943751
	*Lefua echigonia*	16,559	30.7	24.8	16.1	28.4	AB054126
	*Lefua nikkonis*	16,589	29.9	26.3	16.7	27.1	AP011300
	*Oreonectes furcocaudalis*	16,569	31.1	29.5	12.9	26.5	KX778472
	*Oreonectes platycephalus*	16,580	30.2	26.9	16.1	26.8	AP011296
	*Schistura balteata*	16,564	31.7	27.0	15.3	26.0	AB242172
	*Schistura corica*	16,572	29.7	26.6	17.3	26.4	AP011445
	*Schistura fasciolata*	16,560	30.9	26.9	16.2	26.1	KY404236
	*Schistura geisleri*	16,819	30.0	28.2	17.0	24.9	AP013295
	*Schistura jarutanini*	16,594	30.3	28.3	16.9	24.4	AP011307
	*Schistura kaysonei*	16,575	30.6	28.2	16.5	24.8	AP011297
	*Schistura notostigma*	16,568	29.8	27.9	17.1	25.2	AP011308
	*Schistura pridii*	16,576	30.8	28.4	16.3	24.5	AP011443
	*Schistura reticulofasciata*	16,603	30.8	27.7	16.5	25.0	KY379150
	*Schistura scaturigina*	16,585	30.8	27.0	16.4	25.8	KU380330
	*Schistura sikmaiensis*	16,581	33.8	21.1	13.5	31.6	KY379151
	*Triplophysa anterodorsalis*	16,567	27.4	25.7	18.4	28.6	KJ739868
	*Triplophysa bleekeri*	16,568	27.1	25.8	18.5	28.6	JX135578
	*Triplophysa dorsalis*	16,572	26.9	26.1	16.1	30.9	KT241024
	*Triplophysa lixianensis*	16,570	27.8	25.4	18.4	28.5	KT966735
	*Triplophysa orientalis*	16,562	27.4	25.5	18.7	28.5	KJ631323
	*Triplophysa pappenheimi*	16,572	28.2	25.4	18.1	28.3	KY419201
	*Triplophysa robusta*	16,570	28.2	25.3	18.0	28.4	KM406486
	*Triplophysa rosa*	16,585	31.8	25.3	15.6	27.3	JF268621
	*Triplophysa siluroides*	16,574	28.8	25.0	17.5	28.7	KJ781206
	*Triplophysa stenura*	16,569	27.8	25.4	18.4	28.4	KX354975
	*Triplophysa stewarti*	16,567	27.8	25.4	18.4	28.4	KJ631324
	*Triplophysa stoliczkai*	16,571	28.1	25.2	17.9	28.8	JQ663847
	*Triplophysa strauchii*	16,590	28.3	25.4	17.8	28.5	KP297875
	*Triplophysa tenuis*	16,571	27.5	25.7	18.6	28.2	KT224363
	*Triplophysa tibetana*	16,574	26.9	25.6	19.1	28.3	KT224364
	*Triplophysa venusta*	16,574	27.8	26.9	18.4	26.9	KT008666
	*Triplophysa wuweiensis*	16,681	28.0	25.7	18.1	28.2	KT224365
	*Triplophysa xiangxiensis*	16,598	30.8	26.3	16.0	26.8	KT751089
	*Triplophysa xichangensis*	16,570	28.6	25.3	17.6	28.6	KT224366
	*Triplophysa yarkandensis*	16,574	31.9	30.4	17.4	20.3	KP050360
Cyprinidae	*Hemibarbus labeo*	16,612	29.7	27.1	17.2	26.0	DQ347953
	*Hemibarbus longirostris*	16,608	27.7	27.2	18.7	26.3	DQ347952

## 3 RESULTS AND DISCUSSION

### 3.1 Mitochondrial genomic structure and composition

**FIGURE 2:**
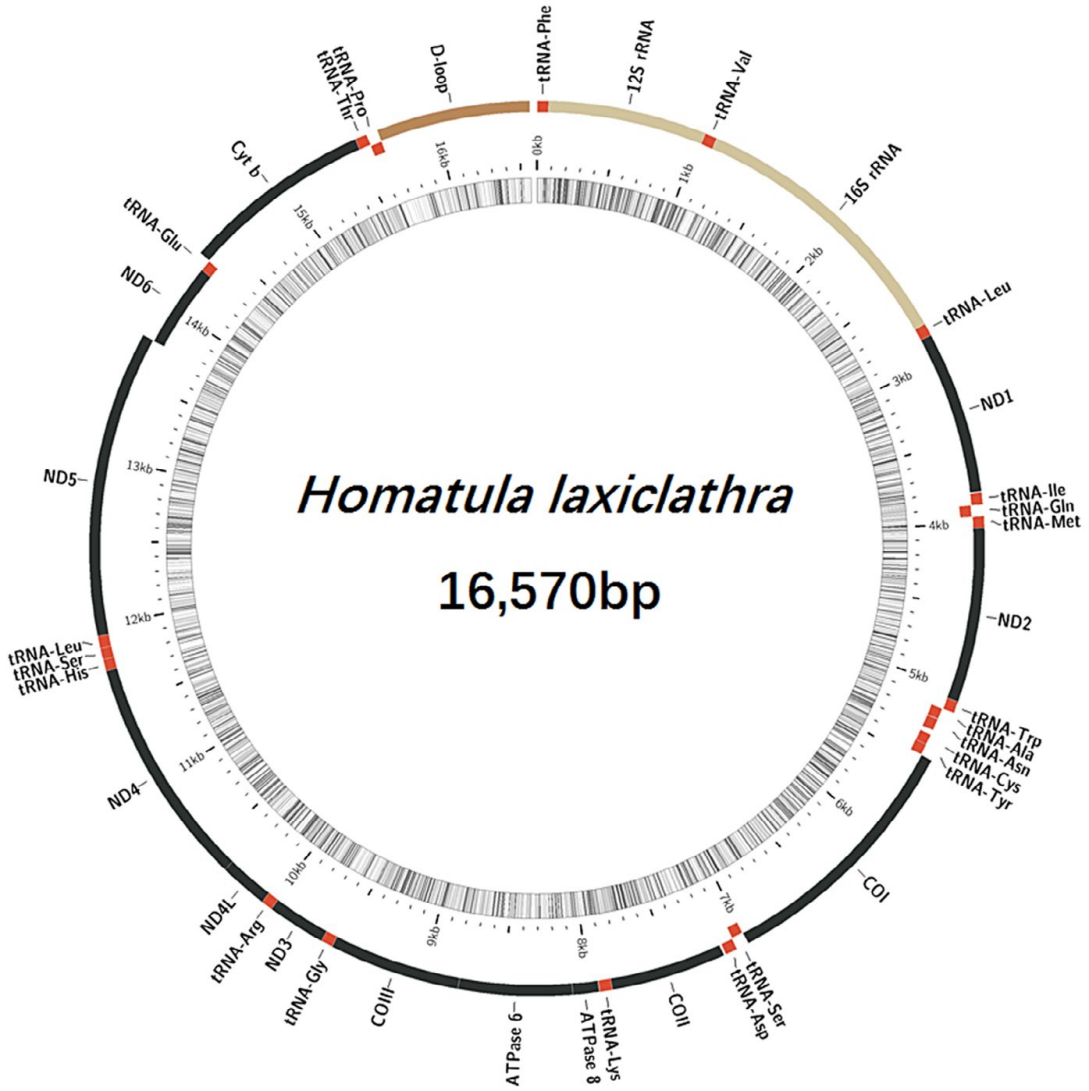
Mitogenome map of *Homatula laxiclathra*, generated from MitoFish (Iwasaki et al., 2013)

The complete mitogenome of *H. laxiclathra* is a circular molecule of 16,570 bp (Figure 2) and is deposited in the GenBank database under accession numbers MK279351. It consists of 37 typical genes, including 13 protein‐coding genes (PCGs), 22 transfer RNA genes, two rRNA genes, and a noncoding region (Table 2). Nearly, all the genes are transcribed on the heavy strand, whereas ND6 and eight tRNA genes are located on the light strand. The structure and composition of *H. laxiclathra* is identical to other mitogenomes of nemacheilids to date (Vlad et al., 2008). The nucleotide composition of the *H. laxiclathra* mitogenome has a gently biased A + T content for 55.7%. The overall base composition of *H. laxiclathra* is the following: A, 29.6%; T, 26.1%; C, 27.0%; G, 17.2%. The overall AT‐ and GC‐skew of *H. laxiclathra* mitogenome are −0.013 and −0.233S. The nucleotide frequency of each protein‐coding gene is A + T > C + G, respectively, showing a strong AT bias (Table 3). For analyses within the genus, the same information from *H. potanini* and *H. variegata* was calculated. *H. potanini* showed the highest A + T frequency at 56.4% with *H. variegata* and *H. laxiclathra* having the most robust AT‐skew. The whole mitogenome base composition of *H. variegata* is highly similar to *H. laxiclathra* with A for 29.5%, T for 26.1%, C for 27.1%, and G for 17.3%, suggesting they share a deep homology.

**TABLE 2:** Annotation of mitochondrial genome of *Homatula laxiclathra*

Gene	Position From‐to	Length (bp)	Intergenic length	Strand	Start codon	Stop condon
tRNA^Phe^	1–69	69		H		
12SrRNA	70–1,020	951	0	H		
tRNA^Val^	1,021–1,092	72	0	H		
16SrRNA	1,093–2,764	1672	0	H		
tRNA^Leu^(UUR)	2,765–2,839	75	0	H		
ND1	2,840–3,814	975	0	H	ATG	TAA
tRNA^Ile^	3,822–3,893	72	7	H		
tRNA^Gln^	3,892–3,962	71	−2	L		
tRNA^Met^	3,964–4,032	69	1	H		
ND2	4,033–5,077	1,045	0	H	ATG	T
tRNA^Trp^	5,078–5,147	70	0	H		
tRNA^Ala^	5,150–5,218	69	2	L		
tRNA^Asn^	5,220–5,292	73	1	L		
tRNA^Cys^	5,323–5,388	66	30	L		
tRNA^Tyr^	5,388–5,456	69	−1	L		
COI	5,458–7,008	1551	1	H	GTG	TAA
tRNA^Ser^(UCN)	7,009–7,079	71	0	L		
tRNA^Asp^	7,083–7,155	73	3	H		
COII	7,169–7,859	691	13	H	ATG	T
tRNA^Lys^	7,860–7,935	76	0	H		
ATP8	7,937–8,104	168	1	H	ATG	TAA
ATP6	8,095–8,777	683	−10	H	ATG	TA
COIII	8,778–9,561	784	0	H	ATG	T
tRNA^Gly^	9,562–9,634	73	0	H		
ND3	9,635–9,983	349	0	H	ATG	T
tRNA^Arg^	9,984–10,053	70	0	H		
ND4L	10,054–10,350	297	0	H	ATG	TAA
ND4	10,344–11,725	1,382	−7	H	ATG	TA
tRNA^His^	11,726–11,795	70	0	H		
tRNA^Ser^(AGY)	11,807–11,862	56	11	H		
tRNA^Leu^(CUN)	11,864–11,936	73	1	H		
ND5	11,937–13,775	1839	0	H	ATG	TAA
ND6	13,772–14,293	522	−4	L	ATG	TAG
tRNA^Glu^	14,294–14,362	69	0	L		
Cytb	14,367–15,507	1,141	4	H	ATG	T
tRNA^Thr^	15,508–15,579	72	0	H		
tRNA^Pro^	15,578–15,647	70	−2	L		
D‐loop	15,648–16,570	923	0	H		

**TABLE 3:** The AT‐ and GC‐skew in the PCGs of *Homatula laxiclathra*

Gene	Nucleotide frequency (%)				A + T (%)	AT‐skew	CG‐skew
	A	T	C	G			
ATP6	28.7	30.2	27.1	14.1	58.9	−0.025	−0.316
ATP8	31.0	26.8	26.2	16.1	57.8	0.073	−0.239
COI	24.6	29.4	25.9	20.2	54.0	−0.089	−0.124
COII	29.2	25.8	28.4	16.6	55.0	0.062	−0.262
COIII	26.8	26.0	28.8	18.4	52.8	0.015	−0.220
ND1	27.9	27.7	29.6	14.8	55.6	0.004	−0.333
ND2	31.3	24.0	31.0	13.7	55.3	0.132	−0.387
ND3	25.2	30.1	27.2	17.5	55.3	−0.089	−0.217
ND4	27.8	26.9	29.2	16.1	54.7	0.016	−0.289
ND4L	22.6	29.0	31.0	17.5	51.6	−0.124	−0.278
ND5	30.1	27.4	28.2	14.4	57.5	0.047	−0.324
ND6	40.2	16.1	30.8	12.8	56.3	0.428	−0.413
Cytb	28.0	28.4	27.4	16.1	56.4	−0.007	−0.260
12SrRNA	29.9	19.9	27.3	22.9	49.8	0.201	−0.088
16SrRNA	35.8	20.5	23.2	20.5	56.3	0.272	−0.062
D‐Loop	32.5	33.6	19.5	14.4	66.1	−0.017	−0.150
Total	27.3	28.1	26.9	16.8	55.8	−0.013	−0.233

### 3.2 Protein‐coding genes

The 13 PCGs of *H. laxiclathra* are similar in component and length to other familial fishes, ranging from 168 bp for ATP8 to 1839 bp for ND5. All these PCGs are coded by the heavy strand except ND6 which is coded by the light strand (Miya & Nishida, 2000). Similar to other loaches, the COI gene has a GTG start codon, whereas other twelve PCGs start with ATG. Five PCGs end with complete termination codon TAA and others with T‐ or TA‐. The total length of 13 PCGs is 11,441 bp, which contain 12 intergenic spacers, the smallest spacer is only 1 bp in size, whereas the longest spacer can be up to 30 bp located between tRNA (Asn) and tRNA (Cys). There are six overlaps ranging from 1 to 10 bp, and the longest region is located between ATP8 and ATP6. Among the 13 protein‐coding genes, ATP6 showed the highest A + T content with 58.9% and COIII at the lowest A + T content with 52.8%.

Codon usage and relative synonymous codon usage (RSCU) of the *H. laxiclathra* mitogenome is summarized (Table 4). Almost all codons are present in *H. laxiclathra* except for the specific mammals stop codons AGA and AGG. The most common amino acids in protein‐coding genes are leucine (463), alanine (331), and threonine (298). Leucine was coded by CUA (213) in *H. laxiclathra* PCGs, the same as in *H. variegata* and *H. potanini*. GCC (147) and GCA (129) are shared equally, coding for alanine, and the same trend is shown by threonine: ACC (120) and ACA (129).

**TABLE 4:** Relative synonymous condon usage (RSCU) in all proteins of *Homatula laxiclathra*

Codon	*n* (RSCU)	Codon	*n* (RSCU)	Codon	*n* (RSCU)	Codon	*n* (RSCU)
UUU(F)	108 (1)	UCU(S)	32 (0.81)	UAU(Y)	41 (0.78)	UGU(C)	8 (0.64)
UUC(F)	108 (1)	UCC(S)	61 (1.55)	UAC(Y)	64 (1.22)	UGC(C)	17 (1.36)
UUA(L)	108 (1.1)	UCA(S)	85 (2.16)	UAA(*)	0 (0)	UGA(W)	89 (1.53)
UUG(L)	16 (0.16)	UCG(S)	7 (0.18)	UAG(*)	0 (0)	UGG(W)	27 (0.47)
CUU(L)	94 (0.96)	CCU(P)	38 (0.73)	CAU(H)	25 (0.48)	CGU(R)	8 (0.45)
CUC(L)	94 (0.96)	CCC(P)	67 (1.28)	CAC(H)	79 (1.52)	CGC(R)	10 (0.56)
CUA(L)	213 (2.18)	CCA(P)	88 (1.68)	CAA(Q)	78 (1.58)	CGA(R)	47 (2.65)
CUG(L)	62 (0.63)	CCG(P)	16 (0.31)	CAG(Q)	21 (0.42)	CGG(R)	6 (0.34)
AUU(I)	175 (1.24)	ACU(T)	35 (0.47)	AAU(N)	41 (0.74)	AGU(S)	8 (0.2)
AUC(I)	107 (0.76)	ACC(T)	120 (1.61)	AAC(N)	70 (1.26)	AGC(S)	43 (1.09)
AUA(M)	118 (1.4)	ACA(T)	129 (1.73)	AAA(K)	61 (1.61)	AGA(*)	0 (0)
AUG(M)	50 (0.6)	ACG(T)	14 (0.19)	AAG(K)	15 (0.39)	AGG(*)	0 (0)
GUU(V)	53 (1.03)	GCU(A)	43 (0.52)	GAU(D)	21 (0.58)	GGU(G)	34 (0.61)
GUC(V)	37 (0.72)	GCC(A)	147 (1.78)	GAC(D)	51 (1.42)	GGC(G)	47 (0.85)
GUA(V)	92 (1.8)	GCA(A)	129 (1.56)	GAA(E)	69 (1.41)	GGA(G)	85 (1.53)
GUG(V)	23 (0.45)	GCG(A)	12 (0.15)	GAG(E)	29 (0.59)	GGG(G)	56 (1.01)

### 3.3 Transfer RNA genes and ribosomal RNA genes

**FIGURE 3:**
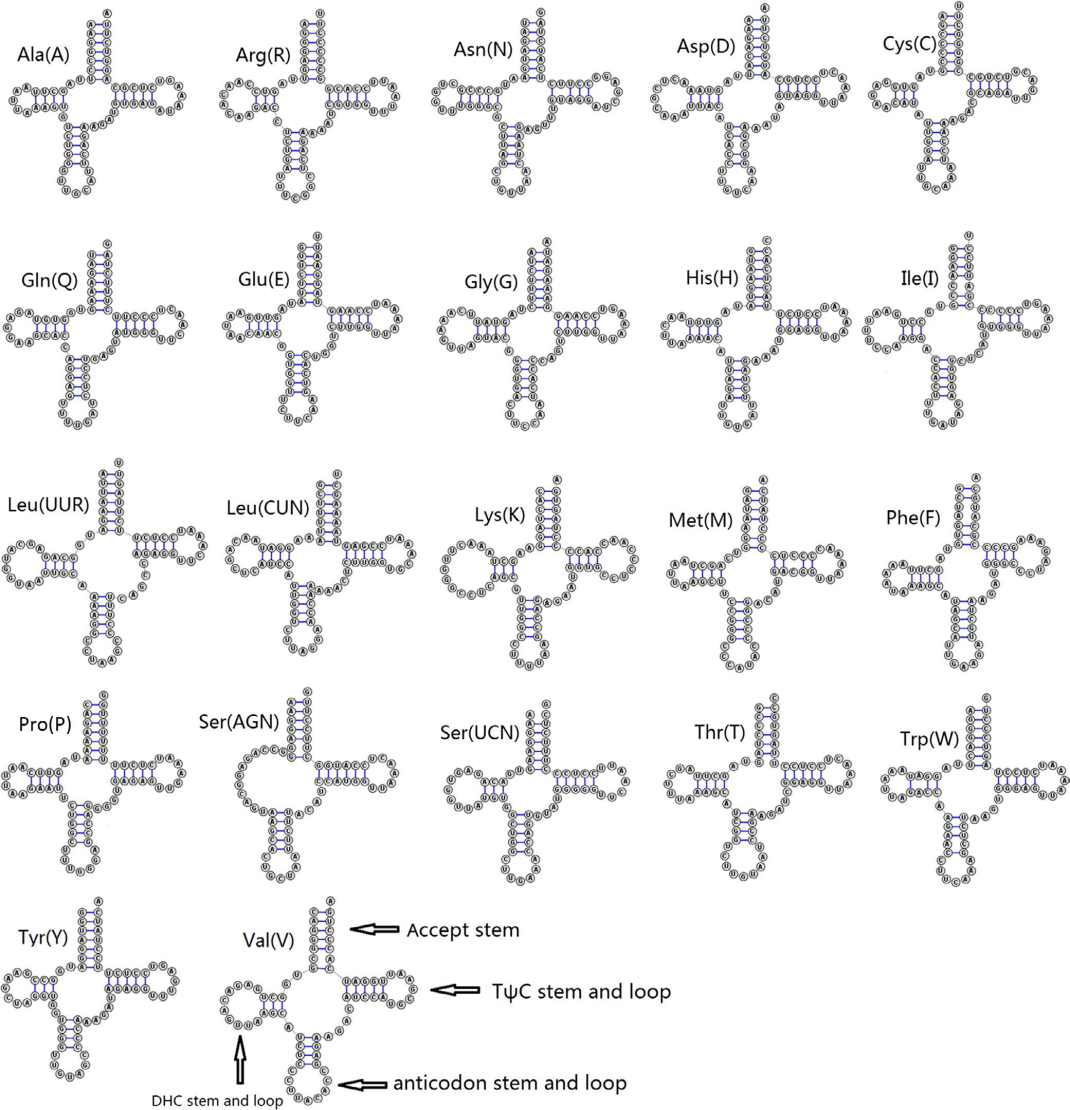
Secondary structures of transfer RNA genes in *Homatula laxiclathra*, generated from RNAstructure 6.1 and SturctureEditor (Mathews, 2014)

All 22 tRNA genes are found in the mitogenome of *H. laxiclathra*. Comparative analysis on potential secondary structures of *H. laxiclathra* tRNAs is shown (Figure 3). Fourteen tRNAs were located on the heavy strand whereas the other tRNAs were on the light strand. The length of all tRNAs was similar, ranging from 56 bp to 75 bp. Nearly, all tRNA genes were predicted to have typical cloverleaf structures, with the exception of tRNA‐Ser (AGN) which lacked a stable DHU stem (Figure 2). This missing stem occurs in most teleost mitogenomes as previously reported (Lee & Kocher, 1995). In addition, some tRNAs showed mismatched pairs in stems (e.g. U‐G and A‐C in the acceptor arm of tRNA‐Arg for three *Homatula* species). These conserved mismatched pairs may be similar to the molecular synapomorphy for the genus. The length of 12S rRNA and 16S rRNA of *H. laxiclathra* were 951 bp and 1672 bp, respectively. The values were similar with *H. potanini* and *H. variegata*, falling well into the size range in fishes. The A + T contents of the 12S rRNA and 16S rRNA of *H. laxiclathra* were 49.8% and 56.3%, respectively, thus indicating some diversity in nucleotide distribution. Both 12S rRNA and 16S rRNA had a positive AT‐skew (0.201 and 0.272), and a negative GC‐skew (−0.088 and −0.062) at the same locations on the heavy strand. Similar to other Nemacheilidae species, 12S rRNA was located between tRNA‐Phe and tRNA‐Val and the 16S rRNA was located between tRNA‐Val and tRNA‐Leu.

### 3.4 Noncoding regions

**FIGURE 4:**
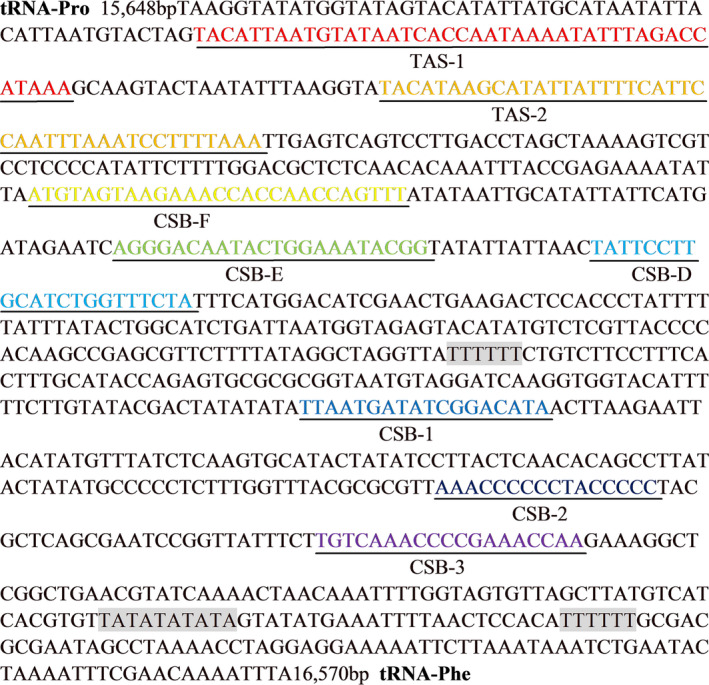
Sequence of D‐loop in *Homatula laxiclathra* with CSBs marked

**FIGURE 5:**
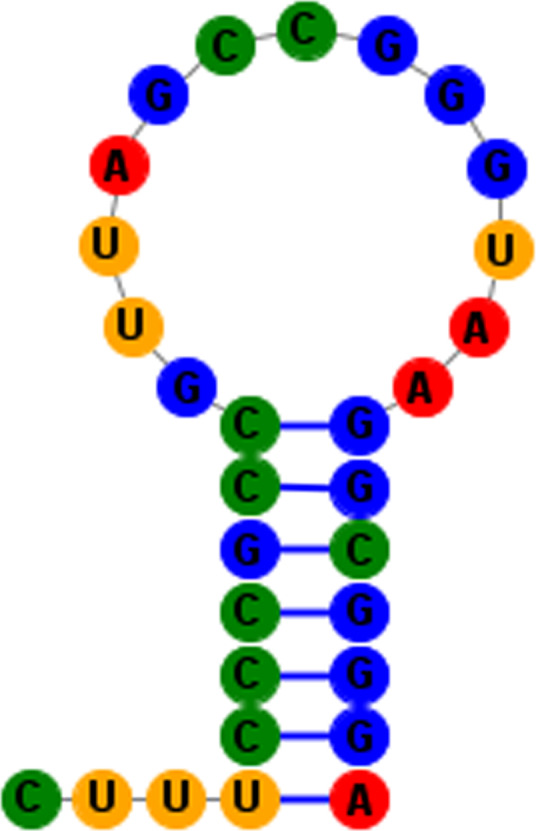
The stem‐loop secondary of O_L_ of *Homatula laxiclathra*, generated from RNAstructure 6.1 and SturctureEditor (Mathews, 2014)

The mitogenome of *H. laxiclathra* has two noncoding regions, the D‐loop and O_L_. The 923 bp D‐loop is located between tRAN‐Pro and tRNA‐Phe with 66.1% A + T content (Figure 4). The O_L_ is 30 bp in length, located in the WANCY region between tRNA‐Asn and tRNA‐Cys with a putative hairpin structure (Figure 5). The D‐loop region is complex and highly variable and can determine the replication pattern of the mitogenome (Liu, Zhang, Tang, Yu, & Zhou, 2010).

By comparing with other Nemacheilidae, the D‐loop can be divided into three functional segments, the termination associated sequence (TAS‐1 and TAS‐2), the central conserved sequence block (CSB‐D, CSB‐E, and CSB‐F) and the conserved sequence block (CSB‐1, CSB‐2, and CSB‐3). The termination associated sequence varies markedly among different lineages, although it can play vital roles in determining the fate of the heavy strand. The core conserved sequence TACAT and complementary sequence ATGTA were detected in TAS, folded into a stable hairpin structure. Two poly‐T stretches and a conserved motif (TA)_5_ were found by comparing against other fishes. Significant tandem repeats were not recognized in the *H. laxiclathra* D‐loop.

### 3.5 Phylogenetic analysis

**FIGURE 6:**
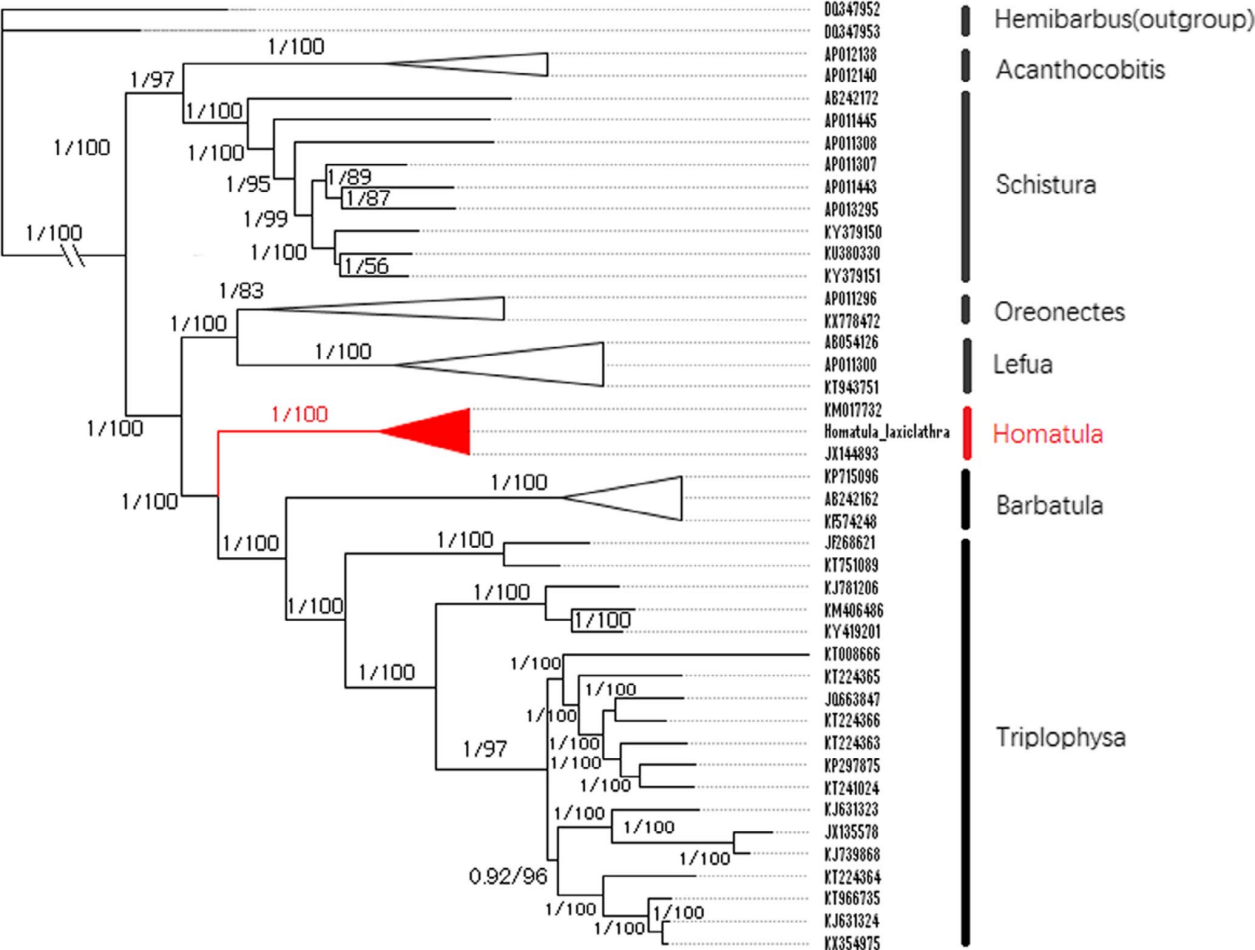
Phylogenetic relationships among Nemacheilidae, generated from MEGA 7 and MrBayes 3.2.7 (Kumar et al., 2016; Ronquist et al., 2012)

Phylogenetic relationships of the Nemacheilidae were reconstructed using two methods, Bayesian inference (BI) and maximum likelihood (ML) (Figure 6). Twelve PCGs from 41 nemacheilid species were concatenated to a matrix and used for phylogenetic analyses; two Cyprinidae species were selected as the outgroups. The phylogenetic trees generated a similar topology that confirmed the findings from a previous study for loach classification (Sgouros, Page, Orlofske, & Jadin, 2019). Both phylogenetic trees consistently showed three major clades, including (I) *Acanthocobitis* and *Schistura*, (II) *Oreonectes* and *Lefua*, (III) *Homatula*, *Barbatula,* and *Triplophysa*. All the congeneric species represented a single cluster for each genus (*Acanthocobitis*, *Homatula*, *Barbatula*, *Lefua*, *Oreonectes*, *Schistura,* and *Triplophysa*), and, the relationship of the Nemacheilidae was consistent with other phylogenetic and morphological studies on these species (Prokofiev, 2010; Stout, Tan, Lemmon, Lemmon, & Armbruster, 2016). Thus, *Homatula* was shown to be valid as an inherent Asian fish group according to where the genus falls out on both trees. Further, *Homatula* shares a close ancestor with *Oreonectes* and *Lefua* making it a sister group. The topology also demonstrated monophyly of three *Lefua* species (Miyazaki et al., 2011). This molecular information provides a more robust data set to support fish classification and identification. In addition, several related articles adapt various standards to classify species, such as phylogeny based on single mitogenome genes or nuclear genes (Liu et al., 2012; Powell, Barker, & Lanyon, 2013; Tang, Liu, Mayden, & Xiong, 2006). Our results are based on the highest coverage of Nemacheilidae mitogenomic data to date and provide an updated view of Nemacheilidae phylogeny.

## 4 CONCLUSIONS

In this study, we present the complete mitogenome of *Homatula laxiclathra* and provide a comparison of this sequence against other *Homatula* species to examine the architecture of mitogenome structure, the location of coding genes, and codon usage. The results integrate updated mitogenomic information of the Nemacheilidae and generate a new phylogeny and relationship among different genera of these fishes. However, many genus‐level taxonomy studies lack robust molecular data and thus the true phylogeny of the loach remains unresolved.

## CONFLICT OF INTEREST

The authors declare that they have no competing interests.

## AUTHOR CONTRIBUTION

**Mengfei Cao:** Data curation (equal); Formal analysis (equal); Investigation (equal); Resources (equal); Software (equal); Writing‐original draft (equal); Writing‐review & editing (equal). **Ling Tang:** Data curation (equal); Investigation (equal); Resources (equal). **Juan Chen:** Data curation (equal); Formal analysis (equal); Investigation (equal); Resources (equal). **Xiaoyu Zhang:** Data curation (equal); Resources (equal). **Russell H. Easy:** Writing‐review & editing (equal). **Ping You:** Conceptualization (equal); Funding acquisition (equal); Investigation (equal); Project administration (equal); Supervision (equal); Writing‐original draft (equal); Writing‐review & editing (equal).

## Data Availability

DNA sequences: The complete mitogenome sequence of *Homatula laxiclathra* was deposited in the GenBank database under accession numbers MK279351. The data have been uploaded into Dryad and available on https://doi.org/10.5061/dryad.nvx0k6dnz.
